# “It’s just crucial to deal with emotions as well as the pain” A qualitative acceptability study of an online emotion regulation skills-focused intervention for people with chronic pain

**DOI:** 10.1016/j.ijchp.2025.100638

**Published:** 2025-10-16

**Authors:** Nell Norman-Nott, Rodrigo R.N. Rizzo, Negin Hesam-Shariati, Jessica Schroeder, Jina Suh, James H. McAuley, Yann Quidé, Sylvia M. Gustin

**Affiliations:** a NeuroRecovery Research Hub, School of Psychology, University of New South Wales, Sydney, Australia; b Centre for Pain IMPACT, Neuroscience Research Australia, Sydney, Australia; c School of Health Sciences, Faculty of Medicine, University of New South Wales, Sydney, Australia; d School of Computer Science and Engineering, University of Washington, United States; e Microsoft Research, Redmond, United States

**Keywords:** Process evaluation, Emotion regulation, Psychological intervention, Digital self-management, Chronic pain

## Abstract

**Objectives:**

Psychological interventions for people with chronic pain increasingly target emotion dysregulation as a contributing factor in psychological comorbidity and pain intensity. The acceptability of these interventions remains uncertain. This qualitative study examined the acceptability of internet-delivered dialectical behavioural therapy for chronic pain (iDBT-Pain), an emotion regulation skills-focused (ERSF) intervention aimed at enhancing emotion dysregulation. iDBT-Pain integrates DBT skills training, and pain science education, in a hybrid guided and self-directed online format.

**Methods:**

We conducted 18 semi-structured interviews with participants enrolled in a Randomised Controlled trial which showed iDBT-Pain significantly improves emotion dysregulation, depression symptoms and pain intensity. Interviews were recorded, transcribed, and deductively analysed according to a theoretical framework of acceptability.

**Results:**

Participants perspectives supported the integration of emotion regulation skills within holistic chronic pain treatment, identifying their efficacy to enhance emotion regulation capabilities and reduce pain intensity. There was also acceptance of the online group-based delivery, and hybrid therapist-guided/self-directed approach.

**Discussion:**

Findings highlight the need for clinical assessment to gauge client readiness for an emotionally focused approach, assess sensitivity to others’ emotions in a group setting, and ensure personalisation of digital components to enhance engagement. These findings have implications for developing iDBT-Pain and for ERSF interventions, particularly those delivered online and to groups. The findings also underscore the role of emotion regulation as a key mechanism in chronic pain, supporting research that advocates for its deeper exploration as a central psychological target in chronic pain mental health treatment.

## Introduction

Emotion regulation refers to our ability to manage our emotional state, to influence the intensity, duration, and frequency of emotions (Gross, 2002). Emerging evidence demonstrates that individuals with chronic pain exhibit a diminished capacity to regulate emotions (Frumer, Harel & Horesh, 2023; Aaron et al., 2020; Lumley et al., 2011; Linton & Shaw, 2011; Linton, 2013), contributing to psychological comorbidity and worsening pain intensity (Lumley et al., 2011; Linton, 2013; Koechlin et al., 2018). A recent systematic review and meta-analysis uncovered that emotion-regulation-skills-focused (ERSF) interventions reduce pain intensity and depressive symptoms compared to usual treatment, and reduce pain interference compared to cognitive behavioural therapy (Norman-Nott et al., 2024).

It is theorised that enhancing emotion regulation transforms cognitive abilities to comprehend and express emotions adaptively and, in doing so, benefits psychological dimensions and pain-related outcomes (Linton & Shaw, 2011). However, measurement of emotion regulation is rarely included in clinical trials of psychological interventions for people with chronic pain (Norman-Nott et al., 2024; Karst, 2024). Hence, while ERSF interventions demonstrate positive benefits for this population, there is limited data to understand whether improvement in emotion regulation underscores the therapeutic benefits and effects on pain-related symptoms.

To address this gap, a recent randomised controlled trial (RCT) investigated internet-delivered dialectical behaviour therapy for chronic pain (iDBT-Pain) (Norman-Nott et al., 2025), an ERSF intervention based on DBT skills training, an evidence-based intervention for emotion dysregulation (Linehan & Wilks, 2015). Building on preliminary trials of DBT for chronic pain (Boersma et al., 2019; Linton & Fruzzetti, 2014; Linton, 2010; Barrett et al., 2021; Sysko, Thorkelson & Szigethy, 2016; Norman-Nott et al., 2022), the iDBT-Pain RCT compared this intervention to treatment-as-usual, prioritising emotion regulation as the primary outcome. It also updated DBT skills for pain-related concerns and integrated pain science education on the mind-body connection and the relationship between emotions and pain (Kang et al., 2021; Butler & Moseley, 2003). Findings revealed significant improvement in emotion regulation at both 9 and 21-weeks, with benefits extending to significant reductions in depression symptoms at both time points and in pain intensity at 21-weeks (Norman-Nott et al., 2025).

While these findings are promising, the acceptability of interventions focusing on emotions rather than pain reduction remains, to our knowledge, understudied in the ERSF literature. A focus on changing emotions may feel invalidating to individuals with chronic pain (Burke, 2019; Driscoll et al., 2021). Therefore, understanding the positive and negative aspects of these approaches may help guide the development of interventions focused on the emotional experience of chronic pain. While our groups pilot study of iDBT-Pain provided preliminary evidence supporting intervention acceptability, the small sample size warranted further investigation to gather more diverse perspectives and enhance generalisability (Norman-Nott et al., 2022). Moreover, incorporating qualitative research into RCTs enhances the understanding of treatment efficacy and may reveal the mechanisms underlying change (Cheng & Metcalfe, 2018; Lewin, Glenton & Oxman, 2009). For example, feedback regarding acceptability of iDBT-Pain may elucidate what contributes or detracts from engagement, and therefore what may determine the effects of the intervention.

The current study aimed to evaluate acceptability of iDBT-Pain with participants in the treatment arm of the iDBT-Pain RCT. We sought to evaluate participants’ commentary, with a key focus to understand the acceptability surrounding, emotion regulation as the target of the intervention, the group-based sessions, and hybrid guided/self-directed internet delivery. Acceptability was defined as the intervention's appropriateness for participants, based on their thoughts and feelings across seven domains: affective attitude, ethicality, intervention coherence, burden, perceived effectiveness, self-efficacy, and opportunity costs (Sekhon, Cartwright & Francis, 2017). Within each domain, barriers and facilitators to engaging in the intervention were explored to identify and inform successful uptake of iDBT-Pain.

## Methods

### Study design

A qualitative research design employing a deductive thematic approach was applied to evaluate participant perceptions of acceptability of iDBT-Pain through semi-structured interviews. A deductive top-down, theory-based approach was chosen to generate detailed information about specific aspects of the intervention specified a-priori (Braun & Clarke, 2006), using the seven domains from the theoretical framework of acceptability (TFA): affective attitude, ethicality, intervention coherence, burden, perceived effectiveness, self-efficacy, and opportunity costs (Sekhon, Cartwright & Francis, 2017). Thus, this study aligns with a post-positivist research paradigm, which supports the use of theory-driven qualitative methods to explore participant experiences within a structured framework (Braun & Clarke, 2025).

This qualitative study was embedded in an RCT of iDBT-Pain (Norman-Nott et al., 2025), registered on the Australian New Zealand Clinical Trials Registry (ACTRN12622000113752) and detailed in a published protocol (Norman-Nott et al., 2023). Ethics approval was obtained from the University of New South Wales Human Ethics Committee (HC220078). The report of this study is guided by the Standards for Reporting Qualitative Research (SRQR) (O’Brien et al., 2014).

### Participants and recruitment

Participants in the iDBT-Pain RCT were adults (≥18 years) who self-identified as currently having chronic pain (pain persisting ≥ 3-months) rated a minimum of 3 out of 10 for the past seven days, without psychotic or personality disorders, or dementia, with access to the internet, fluent in reading and writing English, and living in Australia.

A requirement for participation in this qualitative study was participation in the active arm of the iDBT-Pain RCT. A total of 45 participants were randomised to the intervention arm (Norman-Nott et al., 2025), with the first 24 to complete the intervention invited to provide qualitative feedback. Of these 24, 4 voluntarily withdrew, before the start of intervention (*n* = 2), and after two sessions (*n* = 2) and were uncontactable for an interview. However, where possible details were sought about the intervention experiences up to the point of withdrawal and are included in the results. All remaining participants (*n* = 20) were invited by email a maximum of three times by a member of the research team (NN-N) to arrange a 90-minute semi-structured interview conducted via video conference. A sample size of 15–20 participants was targeted, to attain enough breadth and depth of information (Braun & Clarke, 2021). Written informed consent for participation in this qualitative study was given at the time of consenting to the iDBT-Pain RCT and confirmed verbally prior to the semi-structured interview.

### Research team

The research group included a registered psychologist, professor with PhD, and specialist in chronic pain intervention (SMG), two research fellows with PhD with a focus on chronic pain, applied research, and technological intervention (YQ, NH-S), a physiotherapist with PhD and interest in the study of chronic pain and qualitative research (RRNR), a clinical researcher, psychology graduate and PhD candidate with a background in technology (NN-N), a researcher with PhD and interest in the design and application of technologies for improving mental health (JSu), a user experience researcher with PhD experienced in formative studies investigating technological intervention for mental health (JSc), and a professor with PhD skilled in chronic pain, psychological research, and intervention (JHM). The varied professional backgrounds of the researchers led to diverse reflection during the analysis and interpretation of the qualitative data.

### Patient involvement

We received input from people with chronic pain in our design and development of the intervention. We carefully assessed the burden of the trial on participants with oversite by the trial management group. We intend to disseminate the main results to trial participants, to the public, and to relevant user-led advocacy organisations.

### The iDBT-Pain intervention

The iDBT-Pain intervention utilises evidence-based protocols to train in mindfulness, emotion regulation and distress tolerance skills from DBT according to the DBT Skills Training Manual (Linehan, 2015), and adapted to address chronic pain specific challenges. For example, inspired by Milton Erickson’s approach to pain perception (Erickson, 1967), we adapted the DBT mindfulness techniques to observe pain without judgment. Participants were encouraged to assign a name or colour to their pain, fostering a more objective perspective while engaging in mindfulness-based DBT practices. Emotion regulation and distress tolerance skills were also modified with pain-specific examples via informational videos, such as illustrating how anger can increase pain intensity. Pain science education was also incorporated into the intervention based on findings demonstrating its role in fostering trust, engagement and adherence in digital interventions for chronic illness (Karekla et al., 2019). Pain science education explained the evidence about chronic pain development, the brain's role in emotional processing, and how neuroplasticity, through psychological interventions, can help unlearn pain signals over time (Kang et al., 2021; Butler & Moseley, 2003).

There are three key elements in the delivery of iDBT-Pain: (1) the iDBT-Pain sessions, consisting of eight 90-minute group-based sessions delivered via video conference on Zoom; (2) the iDBT-Pain app, accessed daily on a smart device such as a smartphone; and (3) the iDBT-Pain handbook, a 130-page printed book sent by mail to each participant. Therefore, the iDBT-Pain interventions is a hybrid model of therapist-guided sessions with self-directed skills-based learning which extends a traditional blended model, whereby in-person sessions are supported with self-directed internet-delivered materials (Erbe et al., 2017; Wentzel et al., 2016). This approach was chosen to leverage the benefit of both self-and therapist-guided interventions. Namely, self-directed interventions are related to meaningful changes in chronic pain symptoms (Barlow et al., 2002), potentially through feelings of empowerment to self-manage treatment (Nicholas & Blyth, 2016), while, therapist-guided sessions provide the opportunity to clarify and discuss concepts and can mitigate attrition (Bender et al., 2011).

**Fig. 1:**
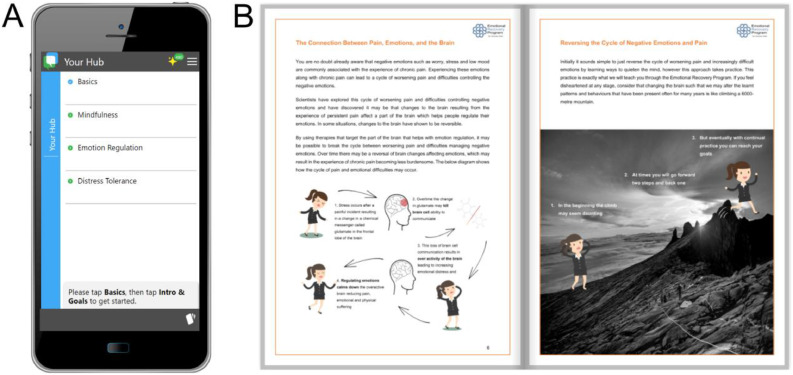
Screenshots of the iDBT-pain app and handbook. A. iDBT-pain app homepage; B. The iDBT-pain handbook showing the role of emotions in the development of chronic pain.

Six of the iDBT-Pain sessions focused on learning the chronic pain tailored DBT skills integrated with the pain science education. Additionally, we included an introductory session in the first week to establish the group environment, and a concluding session in the last week to consolidate the skills learning. Weekly text messages served as reminders for session attendance, and to practice frequently using the app and handbook. If a participant could not attend the iDBT-Pain session live, a video recording of the missed session was provided by secure link. The iDBT-Pain sessions were delivered by a primary therapist, a registered psychologist (SMG), and an assistant therapist, a PhD candidate qualified in DBT skills from the Linehan Institute (NN-N). The therapeutic environment was designed as supportive and nurturing, to provide a sense of inclusion and trust, important to enhance therapeutic outcomes (Furnes, Natvig & Dysvik, 2014), and to enable group discussion useful to facilitate and enhance learning in those with chronic pain (Dysvik & Stephens, 2010). The iDBT-Pain app and handbook allowed participants to self-manage their learning and generalise skills usage to their daily lives (Fig. 1). A multimodal approach integrating both digital and print formats was adopted to leverage the respective benefits of each modality and accommodate diverse participant preferences in engaging with the content. While printed materials allowed for deeper engagement, including note-taking and content highlighting, and helped alleviate eye strain associated with prolonged online engagement, digital resources offered convenience for shorter tasks and facilitated easy access to video content (Johnston & Salaz, 2019). Accordingly, the app which was accessible on participants smart devices, focused on step-by-step tasks to train skills and the printed handbook provided pain science education plus worksheets to practice skills. A full description of the iDBT-Pain intervention is accessible in the publication of the RCT (Norman-Nott et al., 2025) .

### Data collection and interview guide

Data was collected using a semi-structured interview guide (see supplemental files) according to the seven domains of acceptability outlined in the TFA (i.e., affective attitude, burden, ethicality, intervention coherence, opportunity costs, perceived effectiveness, and self-efficacy) (Sekhon, Cartwright & Francis, 2017). The TFA is used to understand how people consider a healthcare intervention to be acceptable, based on their thoughts, feelings, attitudes, and beliefs about an intervention. Closed questions were used to capture participant’s general perceptions about a domain, followed by open-ended questions to elicit further explanation. For example, one of the closed-ended questions exploring self-efficacy asked, “Will you continue to use the skills going forwards?”, which was then followed up with “How confident are you in your abilities to use the skills?”. Prompts were also developed for each question should it be necessary to ask participants to clarify or expand. The interview guide was developed by NN-N alongside RRNR who is knowledgeable about qualitative research and healthcare interventions for chronic pain, and reviewed by SMG who is experienced in the iDBT-Pain intervention and in the development of chronic pain mental health interventions.

All interviews were conducted by NN-N using the video conferencing platform, Zoom, a platform familiar to the participants because they used it during the iDBT-Pain intervention. Video conference was also feasible compared to in-person interviews given that participants were in different locations across Australia. With participant consent, the interviews were recorded, firstly to eliminate interviewer-recall bias, and secondly, it enabled the interviewer to focus on the participant, therefore maintaining appropriate attentiveness (Kelly, 2010). To ensure cybersecurity during the semi-structured interview, access was restricted through the Zoom platform waiting room function, whereby the interviewer granted access to admit the participant into the interview. All interviews lasted from 60 to 90 min, after which, audio recordings were auto transcribed using Otter Pro (Otter, 2023), before being manually checked for accuracy by simultaneously reviewing the audio and written transcripts. Transcriptions were then stripped of any identifiable participant information, assigned a unique identification code, and then imported into NVivo 14 (Lumivero, 2023), a qualitative data management software, for analysis. All recordings and transcriptions were saved on a password protected server accessible only to the researchers involved in the study.

### Data analysis

Data analysis was conducted on the interview transcripts in accordance with a structured approach for thematic analysis (Braun & Clarke, 2006). Data analysis was conducted in parallel with the data collection. This analytic process involved reading and re-reading the transcripts; systematically identifying excerpts that correspond with the pre-defined codes of the TFA and then searching for patterns in the data and organising the data into themes representing the primary perspectives.

The first author (NN-N) completed an in-depth read and re-read of the transcripts to become familiar with the data and to get an overview of each participant’s opinions about iDBT-Pain. Illustrative statements and excerpts from the transcripts were subsequently discussed between the authors (NH-S, NN-N, RRNR, and SMG) before creating a codebook according to the TFA domains (see Table 1), which is useful to improve coding consistency when there are multiple authors’ perspectives (Guest, MacQueen & Namey, 2012). In creating the codebook, we classified “facilitators” or “barriers” to the acceptability of iDBT-Pain under each of the TFA domains. A facilitator was defined as a statement that either improved or, at the very least, did not diminish the perceived acceptability of iDBT-Pain. Conversely, a barrier was identified as a statement that may negatively affect the perceived acceptability of iDBT-Pain.

**Table 1:** Codebook according to the domains of the theoretical framework of acceptability.

TFA Domain/Code	Description
Affective Attitude	How an individual feels about the intervention after participation. Used to discuss participants perspectives about what they liked (facilitator) and did not like (barrier) about the intervention.
Burden	The amount of effort that was required to participate in the intervention. Used to understand whether the participant perceived the requirements of the intervention to be reasonable (facilitator) or not (barrier).
Ethicality	The extent to which an intervention is a good fit with an individual’s value system. Used to enable discussion about what values came through for the participant that resonated well with them (facilitator) compared to any misalignment or concern about values embedded in the intervention (barrier).
Intervention Coherence	The extent to which the participant understands the intervention and how it works. Used to elicit conversation about whether the aims and the methods of the intervention were clear and usable (facilitator) or unclear and need further development (barrier).
Opportunity Costs	The benefits, profits or values that were given up or gained by engaging in the intervention. Used to investigate whether the intervention adds (facilitator) or doesn’t distinguish enough (barrier) when compared to existing treatment/psychological therapies for chronic pain.
Perceived Effectiveness	The extent to which the intervention is perceived to have achieved its intended purpose. Used to discuss the benefits of doing the intervention (facilitator), as well as any perceived shortcomings regarding effects (barrier).
Self-efficacy	The participant's confidence that they can perform the behaviour(s) required to participate in the intervention. Used to discuss whether participants felt they were capable of using the tools provided, and skills taught during the intervention (facilitator) or not (barrier).

*Note.* TFA = theoretical framework of acceptability.

Data were systematically analysed while applying the codebook by NN-N. Two authors (NH-S and YQ) independently scrutinised the first author’s coding and reached a consensus after a discussion with the primary analyst (NN-N). Themes arising from the codes were then summarised in a matrix by NN-N, and scrutinised by RRNR, SMG, YQ and NH-S. This process was iterative to ensure that the interpretations of the themes were credible. Our analysis refrains from emphasizing the frequency of a theme, and instead focuses on the meaning in response to the research question about acceptability (Monrouxe & Rees, 2020). Nevertheless, we incorporate an indication of how frequently participants expressed a similar perspective, utilising terms such as "many," "several", "some," and "a few" in the results (Neale, Miller & West, 2014). Replication of the themes until no new themes were identified was determined to indicate data saturation (Cleary, Horsfall & Hayter, 2014).

## Results

Eighteen of the 20 participants contacted to conduct the interview, responded and agreed to participate. Two did not respond after three approaches via email. The demographic and clinical characteristics of participants included in this study are presented in Table 2. The male/female sex ratio was reflective of the participant sample in the iDBT-Pain clinical trial, including 83.3 % females (*N* = 15) with a median age of 51.5 (range: 27–67) years. The average attendance of the iDBT-Pain sessions was 85.23 %, with participants watching missed iDBT-Pain sessions via video recordings, meaning all received 100 % of the intervention content. Twenty facilitator themes and 15 barrier themes were identified across the seven domains of the TFA. Quotes reflecting responses for each theme within each domain are presented in Table 3. In the text, the quotes are reported using the letter “Q” and the number reported in Table 3 (e.g. Q1 indicates Quote 1).

**Table 2:** Demographic and participant characteristics.

Participant Number	Sex	Age Range	Education	Work Status	Chronic Pain Condition	Income AUD$	% Session attendance^a^
P1	F	45–54	Bachelor’s degree or higher	Employed part-time	Fibromyalgia, Lower back pain, Arthritis pain, Neuropathic pain	$150,000 - $199,999	100 %
P2	F	25–34	Bachelor’s degree or higher	Employed part-time	Headache, Postsurgical pain, Neuropathic pain	$30,000 - $49,999	100 %
P3	F	45–54	Bachelor’s degree or higher	Employed part-time	Lower back pain, Neuropathic pain	$50,000 - $79,999	100 %
P4	M	45–54	Vocational or Similar	Retired	Fibromyalgia, Postsurgical pain, Arthritis pain	$50,000 - $79,999	100 %
P5	F	65+	Vocational or Similar	Not in paid employment	Fibromyalgia	$100,000 - $149,999	100 %
P6	F	25–34	Bachelor’s degree or higher	Not in paid employment	Neuropathic pain	$30,000 - $49,999	75 %
P7	F	55–64	Bachelor’s degree or higher	Retired	Neuropathic pain	$50,000 - $79,999	100 %
P8	F	25–34	Bachelor’s degree or higher	Employed full-time	Fibromyalgia, Postsurgical pain	$80,000 - $99,999	87.5 %
P9	F	55–64	Vocational or Similar	Not in paid employment	Arthritis pain, Neuropathic pain	Less than $30,000	87.5 %
P10	F	55–64	Some University but no degree	Not in paid employment	Arthritis pain, Neuropathic pain	$150,000 - $199,999	100 %
P11	F	65+	Bachelor’s degree or higher	Employed part-time	Neuropathic pain	$50,000 - $79,999	100 %
P12	F	45–54	Vocational or Similar	Employed part-time	Complex Regional Pain Syndrome (CRPS)	Less than $30,000	87.5 %
P13	F	65+	Bachelor’s degree or higher	Employed full-time	Lower back pain, Arthritis pain, Neuropathic pain	$50,000 - $79,999	87.5 %
P14	M	55–64	Vocational or Similar	Not in paid employment	Lower back pain, Cancer pain	$50,000 - $79,999	100 %
P15	M	65+	Secondary	Retired	Fibromyalgia, Neuropathic pain, CRPS	$30,000 - $49,999	100 %
P16	F	25–34	Bachelor’s degree or higher	Employed full-time	Lower back pain, Arthritic pain, Neuropathic pain	More than $200,000	87.5 %
P17	F	25–34	Bachelor’s degree or higher	Employed full-time	Neuropathic pain	More than $200,000	87.5 %
P18	F	35–44	Bachelor’s degree or higher	Employed full-time	Fibromyalgia, Headache, Lower back pain, Neuropathic pain	More than $200,000	62.5 %

*Note.*^a^Participants that missed a zoom session watched a video recording to catch-up on missed content before the next session.

**Table 3:** Quotes from participants.

TFA Domain	Facilitator/Barrier Theme	Quotes	Quote Number
Affective Attitude	Felt validated and less “alone” by connecting with others in the group sessions. (facilitator)	Chronic pain can be really isolating, I actually don't really know anyone else with chronic pain. So it was great to sort of meet others in the community, who also had these issues and hear their perspectives and points of view (P16).	Q1
		I liked the ability to also connect with people that had been through similar things in the past. So the way I describe it to friends is it's like an Alcoholics Anonymous group but for chronic pain………I think that given that I already see a psychologist, one on one, it's great to talk about my perception of pain and what I can do, but having the peer support. That's been really like the one thing I haven't had so far, and actually sitting in a room with other people that are like, wow, I'm actually not the only one. And this person's got, burning in their hand, or this person feels back pain. It's really made me like realise, wow, you're not the only one with this problem. So that's been really valuable, like so valuable…..So I was thinking like, at the end, it was kind of I think that was the hard thing, that we didn't really get a chance to sort of, I don't know, maybe like, even create, a little Facebook support group or something. I think it would be nice to have something where people could opt in voluntarily if they wanted to do that (17).	Q2
		It was really nice to have other people for various other reasons, you know, having chronic pain journeys, and hearing their perspectives and we all kind of seemed to really know what we were talking about. You know, we're all connected in this in this way. So probably I found that the thing that I hadn't had before that was the most helpful (P7).	Q3
		I thought that was good having group sessions. I liked that. I was a bit shy in the beginning, but it was interesting to hear that other people have the same issue as what I have, which sort of showed me it's not just me (P13)	Q4
		Sometimes I feel a bit alienated because I'm quite young. And I get comments like, well, you're young. So how can you have pain and all this, and then I get upset. I like that it wasn't just me that was younger than everyone else (P8).	Q5
		The thing that I really liked about it was the actual Zoom meeting, and talking to people in similar situations (P11)	Q6
Affective Attitude	Felt the skills were communicated in an informative and/or motivational way (facilitator)	I liked the fact that the app was framing the skills in a conversational way. When I first saw it, I thought I'm not sure about this but then I actually did really like that conversational style. I did find it funny how you can trick yourself to find some comfort in having a conversation with a machine. But I did actually like that approach (P18).	Q7
		I like the information videos. They were informative and they weren’t too preachy or scientific. They gave out the information in a very friendly and open way (P9).	Q8
		I liked having the lessons and things to do each week, that kept me motivated, and using the app was a good thing as well (P11)	Q9
		The app was excellent for follow up for anything that was covered each week. And the book, I really liked the booklet because it means I can easily go back and see it. I'm probably a bit more old-school and I like having the book, and it feels like I can flick through it and find something if I need it. Probably more than the app actually, for me, which may be better for younger people more used to always using the phone. So I thought that was great having some modern technology aspects, some group interactive sessions plus the hard copy booklet (P7).	Q10
		The app was a great sort of reminder of the different techniques that we can use. I really loved the group sessions, it was great to see other people in similar conditions, because it can be quite isolating when you've got chronic pain. And it was just also great to see the positive feedback from other people as well. Because yeah, it was just such an amazing programme. I've told so many people about it, and DBT for chronic pain in general (P16).	Q11
		I'd done some work already on DBT, so I was aware of some of the things, but to go through them systematically I thought was really good. (P1).	Q12
		So it was sort of over extended period of time, and even having the breaks that we had during Easter. And all of that I felt was good, because it took time to process what we were being taught and then applying it, and then hearing how everyone else was doing with it. And we were quite a close-knit group in the end so I think that was all really good and helpful (P8).	Q13
Affective Attitude	Gained encouragement and learning from the group. (facilitator)	You could hear other people share their experiences and you shared your experiences around it and it was kind of like, oh yeah. You know, and I think that was beneficial cause you learned through that experiential stuff so much. Not the just skills themselves, but people's experience of the skills (P1).	Q14
		With that group mechanic happening, when people were bringing up how they had used the skills. And how they had helped, that's more useful than just being shown the skill because it widens your perspective immediately. Just like, oh, I hadn't even thought about. We are creatures of habit by nature. So we will apply a skill as we have always applied a skill and to hear a different perspective, and it might just be a tiny change (P4).	Q15
		To see the benefit in other people as well, not just me and how I benefited, but seeing other people, it was so uplifting (P10).	Q16
		Hearing their stories and how they were coping with things. So yeah, really, it was very positive all around (P11).	Q17
		I really enjoyed the group sessions, particularly the group zoom sessions, I thought it was really nice to have that interaction with other people suffering from chronic pain or managing their chronic pain. And I quite looked forward to those group sessions (P7).	Q18
		I was pleasantly surprised in terms of just the style and format and the way that it was presented. And even how my thoughts changed along the way as well. I remember that in the first meeting and sitting down and listening to everyone's stories, and when your time poor, and you kind of listen to everyone and your thinking, what am I going to get out of this and listening to this and listening to that. And then as time went on, and even as soon as the second meeting, I really did begin to see the value. More than, than I expected to (P18).	Q19
Affective Attitude	Preference for more personalised interaction (barrier)	I think I'm more of a person who would like to talk to people rather than the app (P11).	Q20
		If it [the program] is in-person, people do get to interact a little bit more and you can develop closer relationships (P6).	Q21
		I do think that being face to face sometimes is more personal, but also for people with pain and in terms of accessibility, things are more complicated. And even with me, I might not have been able to do it if I had to go somewhere because of work. (P8)	Q22
		It's like, well this isn't for me. This is so generic. It could be for anyone. But they're not dealing with me. And when you are dealing with chronic pain, that kind of internal monologue becomes very self-destructive. Very quickly. Because you've been rejected by society a lot already. Because your limitations they limit you from your ability to go out and be who you were. So, when you are using a product that is supposed to be designed for you. That throws those limitations in your face that's not necessarily a positive thing. So a user profile that alters the questions or options, I think would be terribly useful (P4).	Q23
Affective Attitude	Felt concerned or distressed by other in the group sessions (barrier)	I think it can be tricky, especially when you have some people that have different ideas and mindsets and challenge some ideas and don't understand the concepts and go off in tangents and things. So it's hard to redirect that (P6).	Q24
		When you could see the impact on people, it really did impact on me emotionally and then it caused a bit of, I suppose, distress which I probably didn't recognise at the time, but now, like I'm just kind of thinking about it as we're talking and you know, you have empathy for people, you also have the experience of it as well. And I think that does kind of the more you focus on something like the pain or the things that you've missed out on in your life because of pain or the lack of understanding perhaps from family members or, or friends or whoever which has been experienced by so many people in the group. And then you go, oh yeah, this is actually really significant. And I think that kind of supportive environment is helpful, but it also can be in itself a form of distress because you resonate so closely with the experiences (P1).	Q25
		I found it a little bit overwhelming, but I think for me, sometimes I can take on other people's pain when I hear their experiences (P3).	Q26
		In terms of the program itself, I found it quite difficult to be honest. The Zoom calls part of me appreciated that I could actually connect with other people who might share some of my experiences. But at the same time from an empathetic perspective, it was very difficult. And I found that after each session, I was very drained. And my pain was worse (P2).	Q27
		I'm probably 50/50 on the group setting. Sometimes, you don't need to listen to other people's issues……I get a lot more value when it's one on one, really (P15).	Q28
Ethicality	Felt that the delivery was non-judgemental, compassionate, and authentic. (facilitator)	The way you guys did it, it was just very non-judgmental, it was very inviting. And I think that's the way you've got to approach things. Because chronic pain is a highly debilitating and insidious and stressful time for people and it heightens your emotions. So having compassion and kindness is so important. And I think you guys did that really well (P16).	Q29
		I've always been told you're a type A personality. So you're more inclined to have these things and whatever. But people say that, but then they don't like tell you things to help manage it. So I feel like with this program, it was good, because it was acknowledging that, those things can contribute to pain, but it's not the cause…………Your program was setting realistic expectations, which actually made me engage with it more, rather than people like trying to make all these heightened promises about what’s going to happen………Your program didn't make me feel pressure on myself that if I'm not doing it, right, that it's my fault, because my pain is not gone away. Because I feel like with some of those programs, I feel oh well, they're saying it should go down to zero, and it's not so there's something wrong with me, why can't I do it right. And it would be negative in that way where I just feel really upset and like, it's something wrong that I did but yours didn't make me feel that way at all. So that was good (P8).	Q30
		And I saw in the groups that some people shared stuff and other people said, Yeah, that's a great idea, or Yeah, I really understand you. And I thought that was really good. People were being supportive of one another. I really liked that. (P13).	Q31
		It emotionally, it helps. It gives you a place where not that it’s an outlet, but you know that you’re not going to be judged. That’s in itself is therapeutic (P4).	Q32
Ethicality	Perceived an alignment with beliefs or faith (facilitator)	Buddhism is all about taking responsibility and your effect on the environment. The wisdom within this program is perfectly in accord with my Buddhist practice. There's nothing there that contradicts it. And my Buddhist practice is great for holistic remedies, but the program is narrowing down to the stress pain element of life, which is a big element of my life. So that's why it's important. (P14)	Q33
		As a Christian, I've always believed in having good values and tried to, so a lot of that was a lot of what I thought about anyway. So it kind of really brought it all into focus, which is good. I love the loving kindness meditation and I have been doing that for like 50 years because as a Christian, I pray, so it's, basically doing the same thing. (P5).	Q34
		It's a very good basis to live your life that you don't think about tomorrow, because tomorrow's not really anything and past is just perception. So living in the moment is really the best way forward to live (P11).	Q35
		I wanted something that was a drug free approach to helping manage my mindset. I wasn’t after a miracle cure, or a miracle pill, I wasn’t expecting to do the course and be miraculously better (P17).	Q36
		I do Reiki and meditation anyway. So I’m on that path, if you like. And I do get benefits from that. And your program helped me to develop my own techniques. I do meditation in certain set ways. And I’ve tried to do just regular mindfulness in the past, just in a different way to my usual meditation. So this program has enabled me to explore mindfulness in a different way. Because the books and things like that you read, it’s about be mindful eating and mindfully, this mindfully that. Your program really helped to emphasise that it’s the few minutes here and there. And for me, it was learning to just be mindful, as you were saying, if you’re sitting having a coffee, and you’re just observing people going past, and then your process of observing and describing and participation. You’re kind of immersing yourself in that just for those few minutes (P10).	Q37
Ethicality	Valued an opportunity for learning. (facilitator)	As you started to talk about the changes in the brain, that's when you’re going to get people on board to say oh, it’s a neurological change. It’s not just a psychological thing, or a willingness to get through my pain (P3).	Q38
		Given that I have a psychology degree, I'm doing my psych honours, like the emotional part aligns with what I value, which is, well, if you can't change a situation change how you think about it (P17).	Q39
		I did my A-level psychology in the 80 s, so I do have that interest in the science behind everything. And when you would give out factual statistics without being too scientific, you know that about the research, and the evidence has shown that, you know, if you name an emotion, it can help kind of dial down that emotion when you recognise it and name it. Things like that, for me, are very important (P10)	Q40
		And then having people that were like, of all ages, I felt like they had a lot of wisdom that I could draw from (P8).	Q41
Ethicality	Implication that pain is in the mind. (barrier)	That initial thought about mindfulness and people's like abrasiveness that maybe you're telling me again, that it's in my head. So there's that initial process, I think that people need to work through to get to a point where they're accepting of the mindfulness process, there is a bit of a barrier there. And I think you just got to get people over that hump, to then take a breath and say, okay, and really that whole neurological connection about how mindfulness changes the brain, I think is a really important part of that. (P3).	Q42
Ethicality	Perception that emotion is a “feminine” concept. (barrier)	As an adult male in their fifties, I am going to have default different inclinations for emotional stress to women. And those men in my generation we don't talk, we don't ask for help. You get over that stuff, hopefully. (P4)	Q43
		For men, a male focus would be good, remember, women are more in touch with their feelings, we're allowed to feel whereas a male focus probably would be of benefit (P9).	Q44
Ethicality	Respect for time (barrier)	I think when you have your own personal concerns, I don't want to take up the time of other people with that, generally speaking, and I did find that in the Zoom meetings that I felt sometimes I was saying too much, and not letting other people have a turn (P11).	Q45
		Originally, because life moves really fast for people and you know, everyone’s looking for that instant gratification, like quick let’s get to the point, let’s get to the point. But I think that settled really quickly. For me, I might have thought, oh, gosh, listening to everyone’s stories, and not from an, you know, full empathy and appreciate listening to them. But when you’re in a, you’re just trying to kind of get to the good stuff. You know what I mean, the material, initially I was thinking that, but then I completely reframed and saw the value of it (P18).	Q46
Burden	Found the expectations realistic and achievable. (facilitator)	Even though it was an intensive program, the actual expectation, or the things that you were asking us to do weren’t a burden. It was very realistic (P8).	Q47
		The fact that it wasn't so much content is actually a good thing, because then it was easy to just apply it to my everyday life and wasn’t overwhelming. Whereas something like curable, where it's just like, all of these things, and there's no structure and it's just like all this information is really not straightforward on how to engage with it or how to apply it to your life (P8).	Q48
		You wouldn't want to overload people with information that's difficult to absorb. So I think it was a good amount and like the book is a good size and things like that (P16).	Q49
		I found it okay. But also, I wasn't working, so I didn't have much else on my plate, but I didn't find it too much.	Q50
		I think within the week timeframe there wasn't too much to cover so I think it was Okay. I probably wasn't in the app daily. I'd probably spend a longer timeframe like half an hour, 45 min to an hour on it one day, and then I'd do it probably like two, three times in that week, but longer sessions. (P6). Everyone that I spoke to about it agreed. Both my psychologist, my pain specialist, who is a neurologist, my pain specialists, who is anaesthetist and my physio they all really supported it, and they thought it was really good. And so it just kind of aligned with everything that we've spoken about. Nobody went, oh, goodness, why are you doing this or anything like that, everyone was really, really positive (P17).	Q51
Burden	Perspective that being online helped reduce the burden in terms of time and accessibility. (facilitator)	I think from an accessibility point of view, like for example, when I'm in a really bad pain flare, I can't really drive, I have to use a walking stick. So public transport is difficult, and it's hard to just get up and going. So the fact that it was online on Zoom, allows it to be a lot more accessible…….I think in this post COVID time, everyone is sort of used to this online format. And it makes life easier, especially when you've got chronic pain, and you might not be able to travel and things like that….and then in that way, you're able to have people all over Australia. So I think it was a good medium to host the meetings on (P16).	Q52
		I think in terms of being realistic, [with an online program] you're able to grab a broader audience from around Australia and things like that. And it’s more flexible in terms of what kind of impairments people have (P6).	Q53
		Having the sessions on Zoom was good. Even being able to do it from your phone, like one time I was waiting to go on a ferry and sitting in the car was great. I was forced into retirement when I hurt my back but we didn't use Zoom then. Things have moved so much since. I hear all about other people using zoom so it was good to try it and I think it was excellent (P7).	Q54
		And also from a time perspective, I think that it would be really difficult to be somewhere physically, for the amount of time required to be able to gain that and then all of the stresses around, I guess, travel to and from a destination, I think that, particularly if you're in pain would be difficult. So I think I would actually prefer the online delivery (P18).	Q55
		What would happen though, is that if I do a snippet of a video, because it was such a difficult time, I'd watch some of it and then I'd have to take a break or I'd do something or even if I was listening to some of it on a walk, that's how I found that I was able to get that in and I was in nature at the time, and it was just something I found. I really enjoyed doing it like that (P18).	Q56
		It [being online] makes sure that everyone can be part of it, even if they're having a really bad day, like a lot of the participants were on the lounge, I felt so bad, you know, just had surgery, but they could still log in So there wasn't a barrier to participation. And I know a lot of the clinics, you've got to get off all medication, you've got to go in there three days a week for eight or nine hours. And there's high physical and personal demand. And I actually think that online is a really good way of reducing any barriers (P3).	Q57
		I, have a specially designed chair that I'm sitting in now that helps. Now, if you're going to a physical location, that 90-minute session could become torture……Turning up to a boardroom, sitting in a chair, that might be horribly uncomfortable for you and there are various conditions, like reasons, people having to, to leave for whatever reason and come back for a quick break. (P4).	Q58
		I love the fact that it's on the phone as well in the app. And it's funny, because when I was in the UK, I was then able to look at some of the videos and things. (P10).	Q59
Burden	Perceived that the benefits of the intervention outweighed the effort of participating. (facilitator)	The benefits have definitely outweighed the burden. For over six years I was trying to kind of regulate these emotions in this one particular context and for the first time in my life I couldn't do it. And I was like really surprised. Normally with any issue, I would, you know, pray about it, take it to God and in a month or two, it gets sorted out. The first time I couldn't, and your research really blessed me because you said, okay, there is a glutamine, there's a problem with the stress process. (P5).	Q60
		From my perspective, there was no burden at all. Okay. It was obvious, it is designed to help and that is its purpose. So I can come in and I can benefit from that. As much as I want to participate. If I choose not to participate, I'm not going to benefit. That's my choice. The best thing for me to do would be to leave (P4).	Q61
		I don't think it was a burden at all, I think that you have to be proactive in supporting yourself. And I think initially, you've got to use it a lot in order to get to a place where you can start to use it on your own and do it on your own. And whether that's not having to use the app, but I can wherever I am, go to sit down in a place, and do some deep breathing or some counting. So I can then do that in different spaces that don't actually don't need to use the app. I mean, I could use it on my phone. But sometimes if it's not appropriate to do so you can actually use those tools in different ways (P3).	Q62
Burden	Found fitting the intervention into a busy schedule difficult. (barrier)	When we had the Zoom sessions. That was a bit tricky, because I'm retired now. And my husband's about to retire. But he's semi-retired. So we do get to do a lot of walking and go away a lot. So we certainly had to work our life around the sessions. But we did, and it was fine (P7).	Q63
		I think it's always difficult with homework, particularly when it's emotive or on those kinds of topics to actually be able to find the time to be able to give them your all, but I think that it definitely was still really useful and why I might not have done it every time. The times that I did, it was good enough. But I'm also glad that I have those support materials to refer to when I need to (P18).	Q64
		The only burden I felt was like on myself when I felt bad when I wasn't engaging with the app as much as I could. I did feel guilty in the back of my mind, like I'm wrecking their trial or something like that, if that makes sense. Like I felt like guilty (P8).	Q65
Burden	Experienced frustrations with the app. (barrier)	There, there was one thing that I realised, like some of the things kept repeating. Like there was one about non-judgmental thoughts. The same example would keep coming each time. And then so I thought for some of them, maybe a few more examples would help (P5).	Q66
		I did like the activities in the app. But then I found that when I did them, they were like the same each time sometimes like the thing of the cat, the gif of the cat sleeping or whatever. And then the music was the same. But obviously, I knew that it's like a prototype as well. It's not the finished app. So I could understand that. But sometimes I think that if there were more options on those things, it might have been more engaging (P8).	Q67
		Normally an app on your phone you know, it's more of a thing you just tap into. So it probably it could improve functionality from that perspective and from having notifications or reminders or whatever. But it was, it was really easy to use and go through. (P1).	Q68
		I only found that if I really enjoyed an activity in the app, and then wanted to do that exact same one, again I'd have to go back to answer a few more questions again, to kind of get back into to do the same exercise again. And particularly when you're learning, and you're doing it for the first time and you need to be able to go back easily and try things again (P18).	Q69
		If there's a space on the app for reflection, like a reflection diary kind of journal entry. I think there just needs to be a space for the individual to be able to explain the pain in the situation that they're in………..I wish that I could click on favourites that I could just have like a little toolbar down the side where I had my favourites and so if I had mindfulness, like if I found, say, the breathing or the counting really helpful that I could on the app some way, just click straight into favourites (P3).	Q70
		There was only one problem I had. In my media hub, where I purchase things, they don't stay (P10).	Q71
Opportunity Costs	Appreciated that the intervention required active involvement. (facilitator)	My main reason for wanting to do the trial in the first place is the active participation. I find that most of the pain program that I've done in the past have been quite passive. And so having to actively engage with people and material it was a really big selling point for me, and I feel like that worked really well. And it was what distinguished it, between apps and pain management programs and books versus what you guys offered (P17).	Q72
		I definitely got more from it than when I’ve just say read things on my own or if I would have just read the booklet or done the app on my own. I think all of the elements together in terms of the app, the handbook, the regular meetings, I think together they reinforce one another (P18)	Q73
Opportunity Costs	Perception that the focus on emotion is beneficial and is something that is missing in other interventions. (facilitator)	So for me, the emotional pain has been my struggle, like for over six years. The pain I could cope with, but the emotional pain was harder. I was really glad you were tackling that (P5).	Q74
		I think in cognitive behavioural therapy any focus on emotions is completely lacking. They ignore, they only do the thinking part. And as was said, in the course, that we have emotions, and we don't want to ignore the emotions they are there. And like I've said several times, they just come out of nowhere, I'll be doing something. And it just appears and I have no idea why. Or I wake up in the middle of a night in terror. And it's not because I'm thinking anything. It's at some other unconscious level. Which Cognitive Behaviour does not address. (P13).	Q75
		I've found like in the past, I did some pain, psychological programmes with CBT. But I just didn't click with it. And I just didn't find it very practical for everyday life. But I just really found that your program was really easy to implement (P16).	Q76
		I think, especially about your emotional wellbeing, it's definitely something that is in desperate need. I think there's a lot of talk about medication, and a lot of talk about mobility. But there's very little, I mean, there is a lot of discussion about psychology and emotional wellbeing. But I think there's very little talk about what needs to happen, but not how it needs to happen, and what that looks like. So I think this absolutely just fills that space. (P3).	Q77
		It's important to target emotions, I've said right from day one. It's 50 %, neuropathic pain and 50 % emotional stress or pain, or battle, they're both equally important trying to get past the neuropathic side of it, as well as the emotional and dealing with it day to day, hour to hour. It's very important (P15).	Q78
		I think when you've got more emotional skills, like the ones you gave us, you do relax more, you feel more empowered (P10).	Q79
		I'm easily dysregulated and the physio had even noticed for me that when I'm more emotionally dysregulated, that the physical aches and pains just get worse. And I don't think that there's a huge understanding. But in terms of caregivers and providers across the board, they're not necessarily that holistic and don't necessarily kind of bridge that gap. So if there are treatments like this available to people that stand alone, then that's great because they do complement one another. And I think the more awareness there is, and the more that it's accepted how closely integrated pain and emotions are. It can only be better for people that suffer (P18).	Q80
Opportunity Costs	Thought that online group sessions relieved pressure to communicate (facilitator)	Doing it on Zoom means, little things like, if you want to mute it, if you're not in a mood to talk a lot. You kind of took that into account, as well, as opposed to being, say, maybe in a pain clinic scenario where you get asked questions, and you kind of put on the spot and not everyone's comfortable with that. But ease of access for most people, I think it's a great idea. And let's face it, we're having a bad day, you can do it from your bed, it doesn't really matter where you do it from. I think more people would appreciate it doing it this way. You see lots of people being forced to go back to pain clinics, who've got terrible pain, who can't sit at all, who are then expected to go every day for five days for four hours at a time. Some people just can't do that. So it's great. Excellent (P12).	Q81
		I liked talking to people. But I also think that there's a little bit of the distance is helpful in some way, like not actually being in a room with somebody. It's got that sort of extra layer that makes you that bit more likely to say things that you might not if someone's sitting right next to you (P11).	Q82
		I actually think the online deliveries been perfect in the sense that it's personal enough that you can listen, contribute, be part of something, but then it kind of also removes that one layer of vulnerability in the sense that when you're sitting face to face sometimes it's more difficult when you are talking about something that's emotive or potentially emotive. Online kind of adds a layer of protection….I think that you're less self-conscious in the sense that it's not when it's one on one, and the focus is on you. Sometimes you're thinking a lot about how you're representing yourself, as opposed to just being in the space. So I think that being in a group setting dilute some of that focus (P18).	Q83
Opportunity Costs	Belief that the intervention wasn’t different enough. (barrier)	I was expecting something different. I was hoping for something new. And the topics that were covered was stuff that I already knew. So I was a little bit disappointed, but at the same time, some of the things that I knew and I had forgotten about, or they were a slightly different presentation, and hadn't practised so it was a good reminder and refresher for me to use those skills that maybe I hadn't used and had forgotten about (P13).	Q84
		I think for me some of the ideas weren't new, so I think it might have helped others that if it was new knowledge, because some of the stuff I had already been doing. It's not that I've been taught, it's that I've probably developed over time and strategies already that I didn't probably have a label to, but I already do them every day. I think having been in pain for 11 years, it's hard to not have found or heard about something and being in the profession of physio as well (P6).	Q85
Opportunity Costs	Thought that the intervention would benefit from a smaller group to encourage increased participation and discussion. (barrier)	I just wish some of them [other participants] had said a bit more. I think that having the option of the chat by, you know, typing the things in there, they were happy to do that. But I would have liked to have just heard a bit more from other people. (P11).	Q86
		Maybe smaller groups. Although I really liked the breadth of our group, and probably depends on the group. But usually around about eight is a good group number (P7).	Q87
Opportunity Costs	Put off using the app due to it being more “screen time”. (barrier)	I spend so much time in technology, I try to not do a lot. I do meditation and body scan stuff and whatever, but I try not to do a lot of mindfulness apps generally (P1).	Q88
Perceived Effectiveness	Perceived less pain or pain flare ups (facilitator)	I do go through quite strong pain episodes in the week, I’ll hit eight, nine out of 10, at least four or five times a week at least. And some of those times, I’ve been able to notch it back down to sort of five, six, just by calming the farm (P14).	Q89
		I think generally speaking, it has been less. Because I can say to myself, well, this is now but it’s not going to be tomorrow and I don’t have to worry about tomorrow. It’s just this momentarily. This is the situation. So it helps that I don’t tend to catastrophise and think, oh, no, this is going to be this way forever, sort of thing. (P11)	Q90
		I noticed during the program that my burning pain was less and I had less break through pain (P13).	Q91
		I just noticed that during that time, when I was doing elements of the program and, and participating. My sciatica and my nerve pain, all but completely disappeared for parts of the program. And funnily enough, returned a bit now that I’ve stopped doing as much of the skills, and I can’t say if that is coincidental. But it, it seems to have an effect on my pain (P18).	Q92
Perceived Effectiveness	Saw improvement in their own ability to calm down and cope with negative emotions and stressful situations (facilitator)	I think that sometimes, particularly for me, I was sitting in a place of anger and hostility about my pain situation and what it prohibited me from doing. And I think what this pain programme did was, it just gave me some more tools to be able to respond better to a situation that I couldn’t change. It didn’t mean I could walk better all of a sudden, it didn’t mean that I could run a marathon all of a sudden, but it just changed my mental state and cognition around it. So that I could sort of activate my kind of logical mode rather than sitting in that fear in a flight or fight response (P17).	Q93
		It’s made me more aware of my emotions, and how they affect myself with the pain. But then also how they can affect others. And then therefore, their reaction as well. So it’s been extremely illuminatory (P14).	Q94
		I haven't been in such a good emotional place in years. So my emotions are really good right now. Even though I've still got the pain (P16).	Q95
		I do feel like my mental and emotional state is improving and that hopefully will help to improve the pain in the longer term as well. (P1).	Q96
		Probably my emotions haven’t changed, but my ability to deal with them and register them and recover from them when I’m getting emotional or angry or triggered. Being able to try and stop that process, be able to calm myself down, refocus myself so I don’t escalate the emotions. (P6).	Q97
		The other thing I found really good was those naming emotions worksheets. I found that useful. And I think if I were in a place and something’s really frustrated me, I might do that again, to work it all out because by the end you’ve kind of noticed you’re less frustrated or less angry or whatever, and you’ve worked out what’s triggered you. I’ve definitely used that and even in my head I’ve used it (P7).	Q98
Perceived Effectiveness	Compared to other pain programs it is more effective for chronic pain (facilitator)	I think it puts things together in a bundle. So it would be more effective than other things I have tried, because it just made me more aware that there’s a lot more techniques to use. Rather than before, it’s just sort of focusing on a couple of things here and there. Whereas this program brought it together. (P13).	Q99
		I think in terms of the actual effect, I think this program is more effective than other things I have done, in the sense that it’s something you can use for the rest of your life. And it’s something that because I’m young as well, it’s something that I will always draw back on. Whereas other treatments I’ve had, for example, that seminar I mentioned, I don’t think about that in my day to day. And it obviously didn’t help me because I don’t look back and think about it. But your program I think about it a lot of the times, and I think about it in my day to day, and I think about how I can apply what you taught me. And that means it was effective. And I feel that part of it is how the program ran as well, because it was over such a long period of time that meant it stuck in my head as well. (P8).	Q100
		I feel like it also complemented the work that I’m doing with my psychologist, but I felt that it was more beneficial because it was actually a pain program, whereas my psychologist isn’t a pain psychologist (P17).	Q101
		This approach is more effective compared to other things I’m doing for my pain because it’s given me tools that I’m using regularly. Whereas before I didn’t have anything. In moments where I have flare ups and things like that, I’m now able to use the tools that you gave us. I can at least calm myself down a bit or reframe what I’m thinking. So, yeah, I mean, its excellent (P12).	Q102
		I think this program is more effective than what I have done in the past, because I have been able to talk to somebody rather I’ve just sort of gone along with my own readings. I’ve actually been able to listen and be heard. And that’s helped more (P11).	Q103
		I would say that is program is more effective than things I have done in the past. I could directly compare it to the one I did in London (P10).	Q104
Perceived Effectiveness	Longer program would be more effective to elicit greater changes in pain intensity (barrier)	I felt like program could be longer. I mean, it would be tricky for people to maintain that, you know, space every Friday. So maybe it could have been where you sort of wean off going from every week, and then every fortnight and then every month, or then every three months (P7).	Q105
		Because my pain fluctuates so much. It’s hard to know in the short term I think, but I do believe all those skills that I’m learning are creating, the more I learn about it and the more I read about it. I do think they’re helping me to create a safer state within my own body. And I think that hopefully that translates to less pain because of that connection that we know about the brain and pain and stuff. So I think it’s probably too early to tell (P1).	Q106
Perceived Effectiveness	Pain reduced while doing the skills but returned when not regularly practicing them (barrier)	I’ve not done a lot of the skills recently. So it’s kind of fallen off, as was always going to be the challenge. But I noticed then today, my pains kind of come back. So I was thinking oh, gosh, it’s a lifestyle, isn’t it? And if you’re not spending that time that you need to. But I think that the tools that you’ve presented, allow for both short quick snap back into things where you can really quickly get yourself back on track. So I’m not stressed about it, because I know that okay, well, I’ll just pick up the app and in the next few weeks or so start to get back into it again, and make that time (P18).	Q107
Self-efficacy	Considered that the resources (app, booklet and zoom sessions) were “easy” to use (facilitator)	It was very easy to use. I used it mainly on my computer. Occasionally when I was out somewhere, I had to wait some, then I would use it a little bit on my phone, but mostly so navigating it was easy. And it was a good idea to have those you know, the treats that we get of watching a video if you get those points. That's good motivation in the stars (P5).	Q108
		Using the app has been quite easy from a technology standpoint. I found it very, very easy to use and quite pleasant. Like it's a sort of a fun kind of thing to do rather than a too hard task thing. And I'm not really very good at technology stuff (P11).	Q109
		I was really nervous to start with using Zoom, I thought, oh, this is not going to work and my husband was kind of lurking in the background just in case I was going to fail badly, but it's been very straightforward. So it's all been good (P11).	Q110
		The zoom was super easy. It was interesting how it enabled people to be comfortable and turn their cameras off if they needed to help with their pain or lie down on the couch. So that, I think was really positive for people (P1).	Q111
Self-efficacy	Confident to apply the skills (facilitator)	I feel quite confident I can do the skills. (P11).	Q112
		I'm very confident in doing the skills I've touched on different aspects before and everything and I want to include it in the way I do things as well. So amalgamating it and make what I do already better (P9).	Q113
		I think when you've got more emotional skills, like the ones you gave us, you do relax more, you feel more empowered (P10).	Q114
		One time you sent me a PDF copy of the booklet and then I've just printed out that section and the worksheet that was about naming emotions, and I just do that every few days and write it out. Like before I go to bed that's something that helps if I've had like a bad day or some particularly strong emotion. It's like a good way to kind of get to grips with that (P8).	Q115
		I feel really confident. Even if I take some time away from it and need to revisit it, I think it's almost like a lifelong skill set. (P18).	Q116
Self-efficacy	Will continue to use the skills in the future (facilitator)	I’ve still got the access to the app so I can always just go back to it as I'm building on the way to move forward with it because I do need to do it. It's not that I don't do things every day, but I’m getting better and doing more as I go (P9).	Q117
		I would like to use it [the app] at least every other day maybe. If I can, I would do it every day, at least a little bit of it (P5).	Q118
		I've become a bit more habitual at doing it at certain times. I wouldn't say I always remember to do it at the times that I need at most. But you know, when I'm doing things like taking a spa or in the shower or something like that, like I try and think do some mindfulness practice there. So it's happening every day, even if it's not happening at that crucial point of need (P1).	Q119
		Because of the different materials, the app and the booklet, I feel like I can return to it anytime, and, and go, Okay, I've forgotten a bit about that, but it's easily refreshed (P18).	Q120
		I just like to kind of dip in to the booklet. A few times a week, I will look. And I might just look at one page. Or I might just kind of flick like this and might think, I haven't done that for a while or remind myself of the skills (P10).	Q121
		And I think that if I feel that I'm not doing the right thing that I've always got the resources there. I've always got the booklet and I've got the app still on my phone, and I can go back and read things and check in I suppose with the app too. I like to have that to and for like you have to put a response in rather than just read something and you have to kind of have an interaction. And I think that the interactions are important. (P11).	Q122
Self-efficacy	Perception that more time and/or resources are needed to become confident in applying the skills (barrier)	I think it just wasn't enough time to be able to fully learn all of the skills and to know them. And to use them I'd have to keep learning them via the app, because I just wouldn't know them off the top of my head……I was wondering, in terms of changing something or adding to something, I don't know whether you can do this with an app because I'm not a person that knows how these things work. But I wondered whether because often I'll go all day without really thinking about the moment, like mindfulness or something like that. And if in the app, you could have something that I could programme to say right at 10:20. I'm going to have a few minutes just to myself to do that and get a reminder (P17).	Q123
		Some more tangible or worked examples of different elements. So the emotions, worksheets, and maybe problem solving and like opposite action, like I think, obviously there were examples in the book and that sort of thing. But there were some of the activities I found it was kind of difficult to work through on my own (P2).	Q124
		I needed more time, probably, to really ingrain some of the new really useful tools into my life. I feel like I need more time and have to discipline myself (P7).	Q125
		We could probably spend more time on those skills if you know in the program where you kind of continued in a follow up way, of like how you progressed over time. I think learning them is one thing, but the challenge of implementing it, like any of these things is I think the challenge of implementation is hard for any skills that you use because people kind of drop off or they get easily demotivated by their own lack of like achieving something…. I think that's what's good about the little infographic thing [that I created. It helps me remember] and I gave one to my daughter actually. But it was more, but remembering to use skills in the midst of stuff that's going on (P1).	Q126
		I would have loved some more time, I felt really sad at the conclusion of the program, particularly because I've missed the last two zoom sessions and didn't get to say goodbye. But it's also interesting to see as people learn more how they become more aware, too, and that sometimes you can reflect on that and then apply that to you to your own life too (P18).	Q127
		I don’t know if in the app, whether there’s a way to set it up so that if someone replies in response to one of the prompts, it could link you to an extra resource or linking back to another video or something like that. Just so it didn’t kind of just leave you hanging. I know it’s all kind of it’s a programmed system. So it’s a bit hard, but I found it a little bit difficult a few times where I’m like, Okay, you’re giving me this prompt, but I don’t know how to answer it. But I kind of just had to muddle through to get to the end (P2).	Q128
		I found the labelling of like, the non-judgmental and whatever, I found that difficult. Fitting and slotting it into those, and then you'd say, what does that mean. And I'd have to go and look in my book to answer because I haven't slotted those things into that label (P13).	Q129
		So because chronic pain can really impact your relationships, and social interactions, I found the ones on interpersonal effectiveness really helpful, like the dear man strategy and all of those acronyms. Because, when you're in a bad mood, and the pain takes over, you can lash out and not think straight or say things that you'll regret (P16).	Q130
		Like a little flowchart of how to connect things together in the sessions. Because you know what it's like, when you do anything, reading a chapter in a book during a course like this or anything. You do your chunks. But sometimes you don't put the chunks together to make the whole picture (P10).	Q131
Self-efficacy	Identified that some skills may be unachievable depending on mobility restrictions (barrier)	At the moment, I'm physically and cognitively alert enough to be able to use it. Whether you use it or not, is another question. But yeah, I am able to use it. I can read the book, I can act on the principles. If I was completely bed bound, there's a lot of things in there that I couldn't do (P14).	Q132
Intervention coherence	Understands the role of emotions in worsening pain intensity. (facilitator)	It's just crucial to deal with emotions as well as the pain. It's that element of dealing with the whole person. And not just looking at the symptom of pain. And obviously, you break a bone, it can be fixed and everything else, that's a usual progression. But it's the holistic approach that is just so important with ongoing pain. (P10).	Q133
		When I was first experiencing a lot of pain, and my pain was first getting really bad, I was very heightened, very upset and I was told so much that it was a part of my personality that was causing it, then that made me reject that concept entirely for a long time. But as time has gone on, it's made me realise, it's just a really vicious circle with pain, because obviously, when you have pain, it affects your emotion, and then your emotion gets worse. And that makes your pain worse. But then it's like after you've had it for so long, and it becomes chronic. It's like what came first? And what's causing what now? and then you just get lost in it (P8).	Q134
		The program made me aware of the relationship between pain and emotions. And I see it, I get upset or something and yeah, it comes on. It intensifies. Definitely. I think if you're relaxed, it's less pain and you're much better able to deal with it (P13).	Q135
		I could really understand the science behind what you're doing. And I thought that was good to have that kind of explanation…… I think that targeting more the emotional side of things is definitely the way to go because most of us have, with chronic pain, have come to that kind of acknowledgment that it’s going to be there (P11).	Q136
Intervention coherence	Comprehends the functionality of the skills and tools (app, handbook, and Zoom sessions). (facilitator)	I have a tendency of catastrophizing. And I used to think that if my fear eventuated, then I would just die or not cope. But the reality is that if you imagine the worst thing, like going out to a whiskey bar and falling, and calling the ambulance and whatever, you still kind of go, Well, I've survived through worse than that. And for me, that was one of the most powerful things that no one had in the past actually presented as a tool to me (P17).	Q137
		It was kind of good to learn and be reminded of foundational mindfulness skills and then the progression through from that. I think that the way that it built up from those basic skills and then the emotional regulation side of it. I think that the video with Marsha in the app, I found that they were quite good clear explanations and helped by reinforcing what I read in the book and what had been covered in the zoom session…..The emotion worksheets. I found that, kind of revealing in terms of how I'm processing different situations and I guess making that distinction between objective evaluation versus subjective. And being aware of whether I'm putting a good or bad label on something like I think that's not necessarily a new concept, but it's valuable to remember - the judgments that we place on things (P2).	Q138
		I always thought that mindfulness was more about distraction or breathing through pain. I never really understood that the brain changes when you go through mindfulness, or use those skills. So I didn’t actually understand that mind body connection, from a neurological standpoint (P3).	Q139
		The three different ways of observing, describing and participating, that really made sense. And now I try to use mindfulness in whatever I do. I'm hoping that long-term it'll have a good benefit. Whether I'm walking to put the garbage out or standing at my kitchen window, there's a beautiful view of hills. So I use that constantly to do my mindfulness (P5).	Q140
		I think that somehow it was made more tangible to me. It seemed to be very step by step in the different channels and the different methods that were used. If one thing didn't work on a particular day for you, the next way of explaining it would so I think that and also listening to other people's interpretations was also interesting. I think that this format and the way that it was framed differently, allowed us to kind of digest that information in a different way that I had previously (P18).	Q141
Intervention coherence	Lacking clarity on the concepts and underlying theory, requiring further elucidation. (barrier)	I think people tend to roll their eyes a little bit when they hear mindfulness because people don't want to think that it's that easy. Which is ridiculous. People tend to think of it as hippie dippy. And it's incredible. And I think that for people who are less educated and less understanding about how our brains work and how life affects us it's really hard for them to grasp that simple mindfulness is so effective. And so I think that is the hardest thing about it is helping people understand just how powerful it is and can be towards anything in their lives (P9).	Q142
		I would have liked [the trainer] to explain more about the real mechanics and neurotransmitters and that background material. Although she [the trainer] seemed keen to keep it at a level that everyone is comfortable with, but I guess to allow for people that are really into that detail, provide either maybe an additional session for those that are interested, or just some materials in the handbook to really delve into that side of things (P18).	Q143
		I think that it needed more academic explanations more literature. I just felt like when, I love the technical stuff, I wanted to know how the brain works. I want to understand that I wanted to know what the studies were. And it just felt like it was so dumbed down, that I just, even if I wanted to engage with something, it was just like you just sit in your corner because we can’t talk about that because everybody else like is just at this level. So for me, I found that really frustrating (P17).	Q144
		I really liked the level of detail in the booklet and how it complimented the Zoom slides and the app, but just providing more in-depth content because I really am sort of someone that really likes content heavy stuff (P16).	Q145
		There's a part of me that wishes that there was just a more direct way to address the pain. Even though if I think about it more logically, and realistically, I know quite well that if I have a really emotional day then the next step is going to be that my pain is going to be worse. And so I think that’s when it’s valuable to have this skills and awareness of emotional processing that goes on. I think having that as something that I can keep as a resource and a tool. I think it’s important to take a step back to remember that and recognise that when in the moment I just want the pain to go away (P2).	Q146
		I think at the beginning, there was a lot about the mindfulness. And that was really important to be guided through why mindfulness is important, how it can decrease your pain, how it can make changes in your brain. But I think that's probably where I was a little bit lost just in terms of how my pain fits in there. And I think in the end there was, there was a lot of talk about, like distress and family and radical acceptance. And I think in some ways, some of that would have been really helpful at the beginning (P3).	Q147

*Note.* TFA = theoretical framework of acceptability, *Q* = Question, *P* = Participant.

### Affective attitude

The domain of affective attitude captures participants’ feelings about the iDBT-Pain intervention. Several participants commented that they felt validated, understood, and less alone by connecting with others during the group sessions (Q1–6). Participant 8 said “I get comments like, well, you're young. So how can you have pain … then I get upset. I like that it wasn't just me that was younger than everyone else” (Q5). While Participant 7 mentioned that the group environment was a unique experience, "hearing their perspectives….we’re all connected in this in this way….I found that the thing that I hadn't had before” (Q3). Additionally, several participants liked the content delivery, describing it as either motivational or informative, expressing enjoyment in the variety of mediums to learn the skills and appreciation of the conversational style (Q7–13). Participant 16 indicated an appreciation of the DBT skills saying “it was just such an amazing programme. I've told so many people about it, and DBT for chronic pain in general” (Q11). While Participant 18 commented about the app, “I did find it funny how you can trick yourself to find some comfort in having a conversation with a machine. But I did actually like that approach” (Q7). There were several other mentions that being in a group with others with chronic pain encouraged and enhanced learning (Q14–19), with Participant 1 commenting that they “learned through that experiential stuff…not just the skills themselves, but people's experience of the skills” (Q14).

A few participants highlighted barriers related to affective attitude suggesting that greater opportunities for individualised interaction either with the therapists, or with other participants potentially in-person would have been beneficial (Q20–23). Participant 11 commented “in-person people do get to interact a little bit more and you can develop closer relationships” (Q20). Additionally, some participants, including one that withdrew from the trial, experienced transient levels of distress during the iDBT-Pain sessions over other participants experiences (Q24–28). Participant 1 commented “that kind of supportive environment is helpful, but it also can be in itself a form of distress because you resonate so closely” (Q25), while Participant 3 said “I found it a little bit overwhelming, but I think for me, sometimes I can take on other people's pain when I hear their experiences” (Q26).

### Ethicality

Ethicality refers to the extent that an intervention is a good fit with an individual’s value system. It was commented by some that the delivery of the group sessions was in a non-judgemental, compassionate, and authentic manner (Q29–32). Participant 16 said “The way you guys did it, it was just very non-judgmental, it was very inviting. And I think that's the way you've got to approach things. Because chronic pain is a highly debilitating and insidious and stressful time for people and it heightens your emotions. So having compassion and kindness is so important. And I think you guys did that really well” (Q29). Participant 4 commented on how they valued this approach, “it’s an outlet, but you know that you’re not going to be judged (Q32). That in itself is therapeutic” (Q32). The uniqueness in comparison to other interventions was noted, with Participant 8 commenting “with some of those programs, I feel….why can't I do it right. And it would be negative…where I just feel it's something wrong that I did, but yours didn't make me feel that way…so that was good” (Q30). Participant 13 noted that “people shared stuff, and other people were being supportive” (Q31), indicating the encouraging environment of the group sessions.

Some participants mentioned that the intervention aligned with their beliefs or faith practices (Q33–37). For example, Participant 14 commented “The wisdom within this program is perfectly in accord with my Buddhist practice” (Q33), and Participant 17 described how the intervention met their needs, “I wanted something that was a drug free approach to helping manage my mindset. I wasn’t after a miracle cure, or a miracle pill” (Q36). Additionally, several participants valued the opportunity for learning and focus on the emotional experience of pain (Q38–41). For example, Participant 8 valued the knowledge of others, “having people that were like, of all ages, I felt like they had a lot of wisdom that I could draw from” (Q41). Participant 3 commented on the value of learning new concepts, “As you started to talk about the changes in the brain, that's when you’re going to get people on board to say oh, it’s a neurological change. It’s not just a psychological thing” (Q38), while Participant 17 stated that “the emotional part aligns with what I value” (Q39).

Regarding ethicality barriers, Participant 3 commented “that initial thought about mindfulness…. that maybe you're telling me again, that it's in my head….I think that people need to work through to get to a point where they're accepting of the mindfulness process, there is a bit of a barrier there” (Q42). A few participants (one male and one female sex) commented that the emphasis on emotions might be off-putting for males (Q43–44), because females, as Participant 9 said, “are more in touch with their feelings, we're allowed to feel” (Q44). A few participants highlighted concerns around the interventions respect for their personal time (Q45) and the time of others (Q46), with Participant 11 commenting that “I did find that in the Zoom meetings that I felt sometimes I was saying too much, and not letting other people have a turn (Q45).

### Burden

Burden refers to the amount of effort that is required to participate. Comments from some participants highlighted that the expectations of the intervention were realistic and achievable (Q47–51). Participant 8 said that “it was easy to just apply it to my everyday life and wasn’t overwhelming (Q47), while Participant 17 mentioned, “my psychologist, my pain specialist….and my physio they all really supported it, and they thought it was really good….it just kind of aligned with everything” (Q51). It was further mentioned by several participants, that participating over the internet aided accessibility, allowing participation from almost anywhere which is especially beneficial when living with persistent pain (Q52–59). Participant 4 mentioned “I have a specially designed chair that I'm sitting in now that helps. Now, if you're going to a physical location, that 90-minute session could become torture……Turning up to a boardroom, sitting in a chair, that might be horribly uncomfortable” (Q58). While Participant 7 said “Having the sessions on Zoom was good… being able to do it from your phone, like one time I was waiting to go on a ferry and sitting in the car” (Q54). Comments from a few participants demonstrated that the benefits of participation outweighed any burden (Q60–62), with Participant 4 explaining “there was no burden at all…It was obvious, it is designed to help and that is its purpose. So I can come in and I can benefit from that” (Q61).

On the other hand, a few participants felt that fitting the intervention into their lives was difficult (Q63–65), with Participant 8 saying “I felt bad when I wasn't engaging with the app as much as I could” (Q64). Commentary from several also highlighted issues with the app and a desire for a more personalise experience in the app (Q66–71). For example, Participant 8 commented, “I did like the activities in the app. But then I found that when I did them, they were like the same each time” (Q68). Participant 1 felt that the app “could improve [in] functionality….from having notifications or reminders” to complete the skills (Q68), while Participant 3 said “I think there just needs to be a space for the individual to be able to explain the pain in the situation that they're in….. I think when you're in pain, you feel like you need to explain that and say this is my situation… Just a space for someone to be able to individualise it” (Q70).

### Opportunity costs

The domain of opportunity costs captured the extent that benefits or values may be given up or gained by engaging in the intervention. A few participants commented that the requirement to actively participate in the intervention was an advantage, and distinguished iDBT-Pain from other programs that lack specific direction (Q72–73). For example, Participant 17 said “I find that most of the pain programs that I've done in the past have been quite passive. And so having to actively engage with people and material it was a really big selling point for me, and I feel like that worked really well” (Q73). Furthermore, many participants valued learning emotion regulation skills (Q73–79). Participant 5 mentioned that “the emotional pain has been my struggle, like for over six years. The pain I could cope with, but the emotional pain was harder. I was really glad you were tackling that” (Q74). While Participant 15 said “It's important to target emotions, I've said right from day one” (Q78), and Participant 10 commented “when you've got more emotional skills, like the ones you gave us, you do relax more, you feel more empowered” (Q79).

The focus on emotions also distinguished iDBT-Pain from other interventions. Participant 13 commented, “I think in cognitive behavioural therapy any focus on emotions is completely lacking… they only do the thinking part…..we don't want to ignore the emotions they are there…..they just come out of nowhere, I'll be doing something. And it just appears and I have no idea why” (Q75). Furthermore, comments from a few participants highlighted the value of online sessions (Q81–83). For example, Participant 12 suggested that “more people would appreciate doing it this way. You see lots of people being forced to go back to pain clinics, who've got terrible pain, who can't sit at all, who are then expected to go every day for five days for four hours at a time. Some people just can't do that. So it's great. Excellent” (Q81). While Participant 11 commented that “a little bit of the distance is helpful in some way, like not actually being in a room with somebody (Q82). It's got that sort of extra layer that makes you that bit more likely to say things that you might not if someone's sitting right next to you”, suggesting an advantage over an in-person setting (Q83).

However, a few participants, including one that withdrew from the trial, expressed the view that some of the skills taught during the iDBT-Pain intervention resembled those taught in other interventions (Q84–85). Participant 13 said “the topics that were covered was stuff that I already knew…. I was a little bit disappointed, but at the same time… they were a slightly different presentation… so it was a good reminder and refresher for me to use those skills” (Q84). Additional feedback from a few participants highlighted that the groups could have been smaller in size to encourage greater interaction (Q86–87). Furthermore, Participant 1 raised a concern that the technological nature of the intervention might be a barrier to engagement, “I spend so much time in technology, I try to not do a lot” (Q88).

### Perceived effectiveness

Perceived effectiveness refers to participants' impressions of whether the intervention effectively achieved its intended goals. Commentary from some participants showed a decrease in pain (Q89–92). For example, Participant 14 said “I do go through quite strong pain episodes…I’ll hit eight, nine out of 10, at least four or five times a week… And some of those times, I’ve been able to notch it back down to sort of five, six, just by calming the farm” (Q89). While Participant 18 commented “My sciatica and my nerve pain, all but completely disappeared for parts of the program….it seems to have an effect on my pain” (Q91), and Participant 13 said “I noticed during the program that my burning pain was less, and I had less breakthrough pain” (Q91). Several participants observed enhanced abilities in regulating and coping with negative emotions and living with chronic pain (Q93–98). For example, Participant 17 commented “I was sitting in a place of anger and hostility about my pain situation…what this pain program did was, it just gave me some more tools to be able to respond better to a situation that I couldn’t change” (Q93). While Participant 6 said “my ability to deal with them and register them and recover from them when I’m getting emotional or angry or triggered. Being able to try and stop that process, be able to calm myself down, refocus myself so I don’t escalate the emotions” (Q97). Additionally, several participants commented on the effectiveness of iDBT-Pain compared to other interventions (Q99–104). Participant 12 said “This approach is more effective compared to other things I’m doing for my pain because it’s given me tools that I’m using regularly. Whereas before I didn’t have anything. In moments where I have flare ups and things like that, I’m now able to use the tools that you gave us” (Q102).

A few participants identified the intervention duration as a barrier to effectiveness, commenting that it could have been longer giving more time to learn the skills and evaluate the effects (Q105–106). Participant 7 suggested “maybe it could have been where you sort of wean off going from every week, and then every fortnight and then every month, or then every three months” (Q105). While Participant 1 said “my pain fluctuates so much. It’s hard to know in the short term…I do think they’re helping me to create a safer state within my own body. And I think that hopefully that translates to less pain…..So I think it’s probably too early to tell” (Q106). Additionally, Participant 18 commented on the need to continually practice the skills, “I’ve not done a lot of the skills recently. So it’s kind of fallen off….my pains kind of come back. So I was thinking oh, gosh, it’s a lifestyle, isn’t it?….[but] I’m not stressed about it, because I know…I’ll just pick up the app….to get back into it again, and make that time” (Q107).

### Self-Efficacy

Self-efficacy refers to the participants' belief in their ability to fulfill the requirements of the intervention. It was mentioned by some, that the various components of the intervention (app, handbook, and sessions) were user-friendly (Q108–111). For example, Participant 11 said of the app, “I found it very, very easy to use and quite pleasant. Like it's a sort of a fun kind of thing to do rather than a too hard task thing. And I'm not really very good at technology stuff” (Q109). While Participant 1 commented on the sessions “The zoom was super easy. It was interesting how it enabled people to be comfortable and turn their cameras off if they needed to help with their pain or lie down on the couch. So that, I think was really positive for people” (Q111). Additionally, it was commented by some participants that they could do the skills (Q112–116) and would continue to train in them (Q117–121). Participant 18 said “I feel really confident. Even if I take some time away from it and need to revisit it, I think it's almost like a lifelong skill set” (Q116). While Participant 10 commented about continuing to use the material to practice the skills ongoing, “I just like to kind of dip into the booklet a few times a week…. And I might just look at one page….and might think, I haven't done that for a while or remind myself of the skills” (Q121).

A potential barrier to self-efficacy was commentary indicating that there needed to be either more time, resources, or support (Q123–131). For example, Participant 7 said “to really ingrain some of the new really useful tools into my life. I feel like I need more time” (Q125), while Participant 1 said, “We could probably spend more time on those skills…I think learning them is one thing, but the challenge of implementing it…is hard for any skills that you use” (Q126). While Participant 2 said of the app “I found it a little bit difficult a few times where I’m like, Okay, you’re giving me this prompt, but I don’t know how to answer it. But I kind of just had to muddle through to get to the end” (Q128). Participant 13 said “If there's a space on the app for reflection, like a reflection diary kind of journal entry. I think there just needs to be a space for the individual to be able to explain the pain in the situation that they're in” (Q129). Additionally, Participant 14 noted that mobility issues could result in varying degrees of ability to perform the skills, “If I was completely bed bound, there's a lot of things in there that I couldn't do” (Q132).

### Intervention coherence

The domain of intervention coherence gauges participants' grasp of the intervention and its functioning. Comments from some participants demonstrated that they recognised the role of negative emotions in exacerbating pain severity, indicating an understanding about the underlying concepts guiding the approach employed in iDBT-Pain (Q133–136). For example, Participant 10 commented “It's just crucial to deal with emotions as well as the pain. It's that element of dealing with the whole person. And not just looking at the symptom of pain” (Q133). While Participant 8 said “it's just a really vicious circle with pain, because obviously, when you have pain, it affects your emotion, and then your emotion gets worse. And that makes your pain worse. But then it's like after you've had it for so long, and it becomes chronic. It's like what came first? And what's causing what now?” (Q134). Moreover, some mentioned understanding how the skills and tools operate (Q137–141). For example, Participant 3 commented “I always thought that mindfulness was more about distraction or breathing through pain. I never really understood that the brain changes when you go through mindfulness….So I didn’t actually understand that mind body connection, from a neurological standpoint” (Q140).

On the other hand, several participants noted a potential barrier to their understanding of the intervention (Q142–147). Participant 18 said “I would have liked [the trainer] to explain more about the real mechanics and neurotransmitters and that background material” (Q143), While Participant 3 commented that “at the beginning, there was a lot about mindfulness. And that was really important to be guided through why mindfulness is important, how it can decrease your pain, how it can make changes in your brain. But I think that's probably where I was a little bit lost just in terms of how my pain fits in there” (Q147).

## Discussion

This study aimed to understand the experiences of participants receiving iDBT-Pain, to determine acceptability for people with chronic pain. Using a deductive thematic analysis in accordance with a theoretical framework, we explored participants commentary to identify barriers and facilitators to engaging in the intervention. A key focus was understanding acceptability regarding targeting emotion regulation, as well as acceptability of the group-based sessions and the hybrid guided/self-directed internet delivery. Our findings have implications for developing iDBT-Pain and for other interventions focused on the emotional experience of chronic pain, particularly those that are delivered online and to groups. Recommendations to refine iDBT-Pain are highlighted in Table 4.

**Table 4:** Summary of recommendations for iDBT-pain.

Domain	Description	Recommendations
Affective Attitude	How an individual feels about the intervention.	Continue group Zoom sessions to enhance accessibility, foster connections, mitigate loneliness, and facilitate learning through discussion. Assess the feasibility of implementing online community groups to maintain ongoing connections among group members. Maintain a conversational communication style across Zoom sessions, and in the app. Encourage the sharing of personal experiences and celebrate individual successes in applying the skills as illustrative examples. Additionally, explore how the app can be more personalised to individuals, such as, by pain condition. After the initial introductory session where participants disclose personal details about their pain, acknowledge the potential for feeling empathy towards others' distress. Bring strategies for emotional resilience taught towards the end of iDBT-Pain in earlier to foster distress tolerance skills. Assess the need for one-on-one check-in sessions to offer support if distress is ongoing.
Ethicality	The extent an intervention is a good fit with an individual’s value system.	Maintain the non-judgemental and compassionate environment for the Zoom sessions. For example, acknowledge individual’s contributions to the group discussions, and where necessary take time to pause and review the group chat to respond to comments at regular intervals. Allow time during the sessions to discuss and encourage sharing and examples of integrating skills in everyday life to demonstrate the flexibility of the skills to align with current practices. Be cognisant of sex-based difference surrounding the topic of emotions and emotional expression and consider whether sex-based groups may be necessary to tailor content appropriately.
Burden	The amount of effort that was required to participate in the intervention.	To enable accessibility and flexibility, continue to deliver the intervention on Zoom. Encourage participating in the sessions live but also offer access to an online recording if individuals need to miss a session. Invest in evaluating technical functionality of the app experience to enhance user experience. For example, enable the saving of skills in the app as favourites so they can quickly navigate to them when needed. Monitor the time required to complete the tasks and consider whether participants need the opportunity to have a break if it becomes overwhelming to complete the tasks in the set time. Explore the possibility of developing the app so it is available for download from the app store to improve the user experience and accessibility.
Intervention Coherence	The extent the participant understands the intervention and how it works.	Clearly outline the science and evidence underlying iDBT-Pain. For example, highlight the purpose of learning emotion regulation skills and explain the role of emotions in pain. Consider offering participants access to scientific literature supporting the foundational theories, as individuals with chronic pain frequently seek out articles and scientific research themselves. To enable a thorough and flexible way to consume the intervention content, continue to provide a hybrid approach which includes different modalities (e.g. online sessions, app, and printed handbook). Assess the feasibility of creating more diverse content to explain the skills, such as informational videos and interactive tasks in the app.
Opportunity Costs	The benefits, profits or values that were given up or gained by engaging in the intervention.	Monitor and reinforce the benefits of self-management and highlight the importance of ongoing practice to generalise skills usage in daily life. Differentiate iDBT-Pain from other interventions by clearly articulating the focus on emotion regulation skills and the evidence for this approach. Set expectations with participants that some skills will repeat what they may have learnt prior (e.g. mindfulness) but they are an important grounding for future skills learning. Maintain the use of the chat function in the Zoom sessions to allow flexible participation that is vocal or written and explain how to use both communication methods in the introductory session. Continue to facilitate skills practice in both online environments (Zoom sessions and the app) as well as offline (e.g., printed handbook) to provide participants with options for regulating their screen exposure. Monitor participants to ensure that screen usage is not perceived as excessive, such as offering breaks during the Zoom sessions.
Perceived Effectiveness	The extent the intervention is perceived to have achieved its intended purpose.	Encourage participants to share experiences during sessions to validate instances where they have effectively managed emotions or perceived benefits from applying emotional regulation skills in coping with pain. Investigate whether the intervention requires more time, or one-on-one delivery to enhance and extend the long-term benefits of treatment.
Self-efficacy	The participant's confidence that they can perform the behaviour(s) required in the intervention.	Continue to check-in with participants that they understand the skills and offer help (e.g. via check-in meetings) to those that need additional support to develop confidence. Investigate the optimum size of the group to attain an experience that is personalised but also scales to meet needs. Evolve the materials to incorporate ways that the skills can be tailored for those with restricted mobility (e.g. through virtual reality). Create a concise infographic summarising all the skills to provide participants with a convenient quick reference guide.

### Emotion-regulation skills focused interventions

Participant commentary on affective attitude, ethicality, and burden indicated that emotion regulation skills were well-received and aligned with the needs of the chronic pain population. iDBT-Pain was perceived as effective to improve emotion processing and expression while also reducing pain intensity, reinforcing its acceptability (Sekhon, Cartwright & Francis, 2017). These findings support our clinical trial (Norman-Nott et al., 2025), and broader research linking emotion regulation abilities to psychological and pain-related outcomes (Koechlin et al., 2018; Norman-Nott et al., 2024; Boersma & Flink, 2025; Lumley & Schubiner, 2019), while indicating a need to further explore the mechanistic relationship between pain, emotion regulation and psychological factors (e.g. depression and anxiety) in people with chronic pain.

Despite broad support to target emotions, some participants asserted that emotions may be perceived as feminine, potentially making ERSF interventions less appealing to males. This perception could explain the predominantly female sample in this study and other ERSF trials (Norman-Nott et al., 2024). Gendered norms related to individuals response to pain, stemming from genetics, hormones, and societal expectations may contribute to females perceiving pain as more emotionally driven (Samulowitz et al., 2018), making them more inclined to engage in ERSF interventions. Additionally, a Lancet review reported that women with chronic pain often face greater invalidation (e.g., from healthcare providers) (eClinicalMedicine, 2024), potentially increasing emotion dysregulation and their need for an emotionally focused approach. Nevertheless, both male and female participants comprehended the concepts underlying iDBT-Pain, that emotions and pain are intimately related, and demonstrated confidence in applying the skills. However, pain science education about ERSF interventions appeared fundamental to this comprehension, echoing the literature that individuals with chronic pain want to understand their condition (Bhana et al., 2015) and the interventions they receive (Karekla et al., 2019). For example, demonstrating the rationale behind mindfulness to aid with emotional reactiveness was key to mitigate invalidation of chronic pain, potentially because there may be a perception, that the biomedical aspects of chronic pain are being dismissed when focusing on emotions (Burke, 2019; Driscoll et al., 2021).

Considering these findings, we recommend clinical assessment evaluates individuals’ requirements, particularly to consider the depth of information needed to rationalise the intervention, alongside an evaluation of any preconceived ideas about the emotionality of pain. To aid this, new educational and training initiatives for clinicians may be key in successful translation of ERSF approaches into practice. Additionally, integrating ERSF approaches within a holistic treatment model that also addresses biological and social factors may help prevent feelings of invalidation associated with focusing on the emotional aspects of chronic pain.

### Group-based sessions

Evaluation of participants commentary identified positive responses to the group-based sessions which simultaneously influenced several acceptability domains including, affective attitude, ethicality, and opportunity costs. Consistent with prior studies exploring group environments (Hestmann, Bratås & Grønning, 2023; Andersen et al., 2014; Alldredge, Burlingame & Rosendahl, 2023), including a pilot study investigating an online group intervention (Mariano et al., 2019), the group environment led to feeling validated and socially connected. Participants also appreciated the non-judgmental, compassionate, and supportive culture created by the therapists. Factors understood to encourage active participation and create a nurturing therapeutic alliance (Dysvik & Stephens, 2010).

However, the group environment was not positive for everyone, with commentary from a few, including one that withdrew, highlighting emotional distress upon hearing others talk about their pain and emotions. Thus, while, self-disclosure plays a crucial role in group interventions, enabling problem identification, learning opportunities (Swiller, 2009), and a forum for sharing ideas (Furnes, Natvig & Dysvik, 2014), a group environment may not suit all people with chronic pain. Based on these findings, including an evaluation of whether an individual is suited to a group environment as part of intake assessment appears to be a particularly important for emotionally focused interventions. This evaluation may be all the more necessary when the intervention is online, like iDBT-Pain, because facial cues and body language indicating distress are less apparent compared to in-person environments where these cues are more visible to the attending clinician (Eccleston et al., 2020).

### Hybrid guided/self-directed internet delivery

Participants responses related to the acceptability domains of burden, self-efficacy, and opportunity costs, supported internet-delivery and a hybrid guided/self-directed approach that blended guided video conferencing sessions with the iDBT-Pain app and printed handbook. Consistent with findings from other studies (Mariano et al., 2019; Booth et al., 2022), online sessions enhanced accessibility, thereby reducing intervention burden, and eased feelings of self-consciousness and anxiety associated with group-based in-person interventions. In agreement with other research (Walumbe, Belton & Denneny, 2021), the chat function in the sessions enabled individuals anxious about vocally contributing to still participate. Given frequent comorbid anxiety among individuals with chronic pain, and the role this has in worsening health related symptoms (Asmundson & Katz, 2009), enabling environments that minimise anxiety and supports contributions may be particularly important for engagement, adherence, and treatment outcomes.

Related to the acceptability domain of perceived effectiveness, the effects on pain and emotions required frequent practice. While a few were unable to maintain their practice in the app, it was also noted that continued access to the app and handbook meant skills could be easily picked up again in the future.

These results align with previous research emphasising the impact of empowering individuals with chronic conditions to self-manage treatment (Barlow et al., 2002; Schroeder et al., 2018). However, we caution that some individuals may need more support in learning the skills and implementing them, especially depending on the competing demands for time, such as work, family and other responsibilities. Relatedly, it was commented that more personalisation in the app (e.g. by pain condition), would encourage engagement, and skills learning. These findings align with the literature that personalisation enables individuals to access content most relevant for them, in turn driving greater engagement and intervention efficacy (Borghouts et al., 2021; Schroeder et al., 2020). Considering these findings, a hybrid approach incorporating both guided and self-directed elements, appears to be appropriate for delivery of an ERSF intervention for people with chronic pain. Although, we caution that some individuals may need more than eight weeks to complete the training, and therefore intervention delivery may be spread out over a longer time span.

### Strengths and limitations

This study benefits from a robust methodology, including a pre-published protocol, a structured interview guide to address key domains outlined in a standard framework for evaluating acceptability, and a rigorous transcription process. This process involved thorough review and analysis of interview transcripts by at least two authors. The interviews took place within a six-week timeframe following the intervention, maximising participants' ability to recall their experiences accurately. However, there are some limitations. Of the 24 participants invited to provide qualitative feedback, four withdrew from the RCT, and were therefore uncontactable for the semi-structured interview for the current acceptability study. However, two of these four participants did provide unstructured feedback following withdrawal and their critical commentary is noted in the results and discussion. We did not examine whether participants’ responses differed according to their level of improvement on the primary outcome of the trial, potentially limiting our ability to explore how treatment effects influences acceptability. Moreover, the semi-structured interviews were conducted by someone familiar to the participants from the intervention (i.e., NN-N) which may have influenced participants willingness to share critical feedback. Nevertheless, all participants shared a range of both positive and negative feedback, including feedback about the therapists and therapeutic environment which reached a critical saturation point where no new themes arose (Braun & Clarke, 2021).

### Conclusions and clinical implications

We evaluated the acceptability of iDBT-Pain, an intervention that demonstrated efficacy to improve emotion dysregulation, depression and pain intensity in a recent RCT for people with chronic pain (Norman-Nott et al., 2025). Feedback from participants was categorised as barriers or facilitators within the domains of a theoretical framework of acceptability (Sekhon, Cartwright & Francis, 2017). Response patterns demonstrated acceptability of an emotion regulation focused approach within a holistic treatment model for chronic pain, whilst highlighting the need that clinical assessment should evaluate participants readiness for an approach that centres on the emotional experience of chronic pain. Perspectives about a group-based approach demonstrated acceptance, and reinforced critical benefits in sharing experiences and validation whilst also indicating the necessity to evaluate an individual’s vulnerability to picking up on the emotionality of others in the group. Participant’s commentary indicated acceptance of an internet delivered approach including a dynamic blend of self-directed learning via digital and printed materials alongside guided online sessions, whilst highlighting the potential opportunities to improve personalisation. These findings have implications for developing iDBT-Pain and for other interventions focused on the emotional experience of chronic pain, particularly those that are delivered online and to groups. The iDBT-Pain intervention is currently being updated based on participant feedback, and these revisions will be further evaluated in future studies. This work will also allow exploration of how treatment response may relate to participants’ perceptions of acceptability. Our findings also highlight the potential importance of emotion regulation as a mechanism in chronic pain, contributing to the research championing deeper investigation into emotion regulation as a central psychological target in chronic pain mental health treatment.

## Authors’ contributions

NN-N and SMG conceptualised the idea for this study. The methodology, analysis plan, interview guide and codebook were developed by NN-N, SMG, YQ, NH-S, and RRNR. Data collection was performed by NN-N, analysed by NN-N, SMG, YQ, NH-S, and RRNR, and critiqued by JHM, JSu, and JSh. NN-N drafted the manuscript. All authors critically reviewed and revised the manuscript for important intellectual content. Successive drafts received substantial contributions from all authors to revise and critically review all content. The final version of the manuscript was approved by all authors. All authors agree to be accountable for all aspects of the work, and to ensure that questions related to the accuracy or integrity of any part of the work are appropriately investigated and resolved.

## Funding statement

This work was supported by the Medical Research Future Fund (Grant ID: MRF2027056). SMG was supported by a Rebecca Cooper Fellowship from the Rebecca L. Cooper Medical Research Foundation. NN-N was supported by the Australian Government Research Training Program (RTP) Scholarship (administered by the University of New South Wales), a supplementary scholarship administered by Neuroscience Research Australia (NeuRA) and the PhD Pearl Award from NeuRA. NH-S was supported by a grant from the National Health and Medical Research Council of Australia (ID2001653). RRNR was supported by the University of New South Wales School of Medical Sciences Postgraduate Research Scholarship and a NeuRA Ph.D. Candidature Supplementary Scholarship. The funding bodies had no role in the decision to publish this research.

## Data availability

The qualitative data generated during the current study are available from the corresponding author on reasonable request.

## Declaration of competing interest

The authors declare the following financial interests/personal relationships which may be considered as potential competing interests: Dr Jina Suh reports a relationship with Microsoft Research that includes: consulting or advisory. Prof Sylvia Gustin reports a relationship with Rebecca L Cooper Medical Research Foundation that includes: funding grants. Dr Rodrigo Rizzo reports a relationship with University of New South Wales School of Medical Sciences that includes: funding grants. Prof James McAuley reports a relationship with National Health and Medical Research Council of Australia that includes: funding grants. Prof James McAuley reports a relationship with Australian Government Medical Research Future Fund that includes: funding grants. Prof Sylvia Gustin reports a relationship with Australian Government Medical Research Future Fund that includes: funding grants. Dr Nell Norman-Nott reports a relationship with Australian Government Medical Research Future Fund that includes: employment and funding grants. Dr Negin Hesam-Shariati reports a relationship with National Health and Medical Research Council of Australia that includes: funding grants. Dr Nell Norman-Nott reports a relationship with Australian Government Research Training Program Scholarship that includes: funding grants. All authors declare no other additional competing interests. If there are other authors, they declare that they have no known competing financial interests or personal relationships that could have appeared to influence the work reported in this paper.

## Appendix Supplementary materials

Image, application 1

Image, application 2
