# Supplementary material for: “It’s just crucial to deal with emotions as well as the pain” A qualitative acceptability study of an online emotion regulation skills-focused intervention for people with chronic pain
Source: Int J Clin Health Psychol. 2025 Oct 16;25(4):100638. doi: 10.1016/j.ijchp.2025.100638 (PMC12552944; doi:10.1016/j.ijchp.2025.100638)
Supplement: Supplementary file 2 [file mmc2.pdf]

iDBT-Pain Semi-Structure Interview Questions

1. What are your thoughts about the program you have received?
2. What aspects of the program did you find most useful/valuable?/ Why?
3. Did you find any aspects of the program unnecessary or less necessary?/ Why?
4. Do you feel this program compliments other methods of treatment that you are using to manage your pain?
5. Have you noticed an effect or change in your pain intensity?
6. Have you noticed an effect or change in your ability to regulate your emotions?
7. How does this approach to lessening your pain compare to other treatments that you have tried in the past? Has it had more effect/less, similar/ different?
8. How confident are you in your abilities to use the intervention (sessions and web app)?
9. Have you had to give up anything that is of benefit to you to do the intervention?
10. Has the effort required to participate in the intervention outweighed the burden of participating?
11. Is there anything missing from this program?
12. What's been your experience of using the web app from a technology standpoint?
13. What's been your experience of the zoom sessions from a technology standpoint?
14. Is there anything that can be improved from a technology standpoint to make it easier for people to use the app and zoom sessions?
15. What's been the most useful skill that you have learnt?
16. Can you give an example of when you have used this skill?
17. Will you continue to use the skills we've taught you?
18. How confident are you in your abilities to use the skills?
19. Will you continue to access the app to train in the skills?
20. How regularly do you think you'll use the app going forward?
21. Will you continue to use the booklet to train in the skills?

22. How regularly do you think you'll use the booklet going forward?
23. What do you think generally about treatments for chronic pain intended to target the emotions instead of targeting only the physical aspects of pain? Has your perspective changed following this program?
24. What are your thoughts on the skills training being online? Have you liked it being online/or not?
25. What do you think of the zoom sessions being group sessions? Do you see advantages and disadvantages to having group sessions compared to one-on-one sessions?
26. On a scale of 1 to 10 how likely would you be to recommend this skills training to someone else with chronic pain?
27. Is there anything else you would like to add about your experience of this program that we haven’t covered already?
